# Resonant Drive Techniques for Electrostatic Microelectromechanical Systems (MEMS): A Comparative Study

**DOI:** 10.3390/s25061719

**Published:** 2025-03-10

**Authors:** Rana Abdelrahman, Alaaeldin Elhady, Yasser S. Shama, Mohamed Abdelrahman, Alexis Jollivet, Dogu Ozyigit, Mustafa Yavuz, Eihab M. Abdel-Rahman

**Affiliations:** 1 Systems Design Engineering, University of Waterloo, 200 University Ave. W, Waterloo, ON N2L 3G1, Canadays2shama@uwaterloo.ca (Y.S.S.);; 2 Waterloo Institute for Nanotechnology (WIN), University of Waterloo, Waterloo, ON N2L 3G1, Canadamustafa.yavuz@uwaterloo.ca (M.Y.); 3 Mechanical Engineering, Benha Faculty of Engineering, Benha University, Benha 13511, Egypt; 4 Electronics and Digital Technology Department, Polytech Nantes, University of Nantes, 44300 Nantes, France; alexis.jollivet@gmail.com; 5 Mechanical and Mechatronics Engineering Department, University of Waterloo, Waterloo, ON N2L 3G1, Canada

**Keywords:** electrostatic MEMS, NEMS, resonant drive, resonance matching, multi-frequency, amplitude modulation, voltage amplification, sensors, actuators

## Abstract

Electrostatic actuation is widely employed in microelectromechanical systems (MEMS) due to its distinct advantages. However, it requires high voltage, typically provided by a power supply and a high voltage amplifier, which is limited in gain, especially at high frequencies. Various methods have been proposed to amplify the voltage signal fed into the system by coupling it in series to an LC tank circuit. In this work, we analyze and compare three methods, resonance matching, multi-frequency excitation, and amplitude modulation. We also compare their performance to that of a voltage amplifier. We demonstrate that resonant circuits significantly enhance performance, offering a more effective solution for high-frequency MEMS actuation.

## 1. Introduction

Microelectromechanical systems (MEMS) have transformed modern sensing and actuation technologies due to their compact size, lightweight design, high accuracy, and cost efficiency. Over the past two decades, MEMS devices have consistently excelled, delivering real-time operation and improved portability [1,2,3]. Their versatility has led to a wide range of applications, including micromirrors [4], sensors [5,6,7,8,9], RF switches [10], and energy harvesters [11], solidifying their role in advanced technological systems.

Among the various actuation mechanisms employed in MEMS, electrostatic transduction stands out for its simplicity, ease of fabrication and integration with electrical circuits, rapid response, and low power consumption [12,13,14]. With the growing demand for high-sensitivity sensors, researchers have increasingly focused on higher-frequency MEMS, exploring their higher vibration modes [15] and studying nonlinear phenomena [16,17]. However, despite its many advantages, electrostatic actuation often requires high operating voltages, posing a significant challenge, particularly at higher frequencies where conventional voltage amplifiers exhibit limited gain.

To overcome the high voltage requirement and amplifier limitations at high frequencies, researchers have proposed various methods to amplify the voltage signal fed into the system by coupling it in series with an LC tank circuit formed by introducing an external inductor *L*. These methods are based on utilizing the electrical quality factor $Q_{e}$ of an RLC circuit driven at its natural frequency $f_{e}$ to amplify the actuation voltage. Common approaches include using resonant circuits [18,19,20], frequency-modulated (FM) [21] and amplitude-modulated (AM) signals [22,23], matching electrical and mechanical resonances [24,25], and excitation using multi-frequency signals [26,27,28,29].

The concept of using resonant circuits dates back to Cady’s pioneering work on piezoelectric resonators [24], where he demonstrated that connecting a quartz crystal resonator to an LC tank can maximize its response. Recent studies have extended this approach to electrostatic MEMS. For instance, Truitt et al. [25] explored resonance matching as a method to improve the readout sensitivity in nanomechanical resonators. Chung et al. [23] employed amplitude modulation to configure MEMS devices as demodulators of AM/FM signals. Jaber et al. [28] and Ouakad et al. [29] investigated the use of LC tank circuits to amplify MEMS responses without external amplifiers. While these studies have demonstrated the effectiveness of each technique, no one has yet carried out a comprehensive comparative analysis among them to elucidate their relative merits.

In this work, we carry out a systematic comparison among those amplification techniques, namely resonance matching, multi-frequency excitation, and amplitude modulation, relative to each other and to a voltage amplifier, which serves as a baseline for evaluation. This study aims to identify the most effective among these methods for MEMS actuation, particularly in high-frequency applications (above 1 MHz), and their limitations. We identify and highlight the advantages of different resonant drive techniques in terms of voltage amplification and tuning flexibility.

## 2. Electrostatic MEMS Actuator

### 2.1. Actuator Design

**Figure 1:**
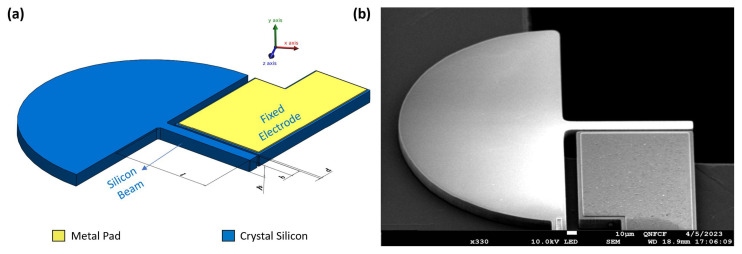
(**a**) Schematic diagram of the actuator. (**b**) Scanning Electron Microscope (SEM) image of the actuator.

The electrostatic MEMS actuator under study is an in-plane actuator composed of a stationary silicon electrode with a metal pad on top made of aluminum and a movable silicon microbeam, shown in Figure 1. The actuator was fabricated using the PiezoMUMPs [30] process. The substrate underneath the beam was fully etched during the fabrication. The as-designed dimensions of the actuator are listed in Table 1.

### 2.2. Lumped-Element Model

The proposed model studies the response of the first out-of-plane bending mode of the microbeam, which is actuated via the (weak) fringing electrostatic field. The model incorporates both electrical and mechanical subsystems represented as lumped parameters. The mechanical subsystem is an electrostatic MEMS actuator modeled as a simple parallel plate capacitor electrically excited by a force consisting of a static voltage $V_{\mathrm{DC}}$ and a time-varying voltage $V_{\mathrm{AC}}$, while the electrical subsystem is an LC tank constituted by introducing an external inductor *L* connected in series with the MEMS actuator.

**Figure 2:**
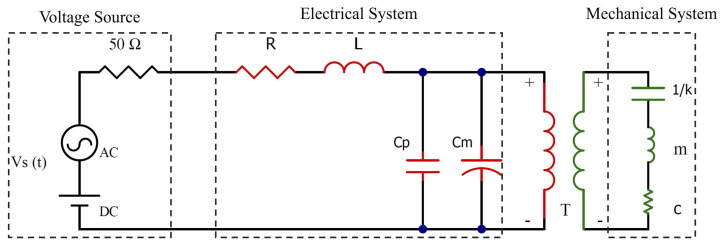
Equivalent circuit of the electromechanical system.

The equivalent circuit of the electromechanical system is presented in Figure 2, with the 50 Ω resistor representing the internal resistance of the voltage source. The electrical characteristics of the system are represented by a series RLC circuit, where *R* represents the circuit’s parasitic resistance, *L* is the external inductor, and *C* represents the total capacitance of the system, consisting of the MEMS variable capacitance $C_{m}$ parallel to the parasitic capacitance in the system $C_{p}$ due to coupling with other structures on the chip and wire bonding. The transformer *T* represents the nonlinearities in the system and the energy flow between the electrical and mechanical domains. The mechanical parameters of the system are represented in an electrical analogy, where the capacitor $\frac{1}{k}$ represents the reciprocal of the mechanical stiffness, the inductor *m* refers to the system’s effective mass, and the resistor *c* represents the mechanical damping. The beam dynamics is governed by the following linear equation

(1) $$\ddot{w}+\frac{\omega_{m}}{Q_{m}}\;\dot{w}+\omega_{m}^{2}\;w=\frac{\epsilon \beta lV{\left(t\right)}^{2}}{2md}\;\mathrm{sgn}\left(w_{\circ }-w\right)$$

where *w* is the out-of-plane displacement in the *y*-direction, $\omega_{m}=\sqrt{\frac{k}{m}}$ is the undamped angular natural frequency of the uncoupled mechanical subsystem and $Q_{m}$ is its quality factor, $\epsilon =\epsilon_{\circ }\epsilon_{r}$, $\epsilon_{\circ }$ is the permittivity of free space, $\epsilon_{r}$ is the permittivity of the medium (air), V(t) is the voltage across the MEMS, and sgn(w) is the signum function. We assume that the top surface of the beam is initially parallel to the top surface of the metal pad on top of the stationary electrode and $w_{\circ }=0.7$ µm is the metallization thickness as measured using a white light profilometer.

**Figure 3:**
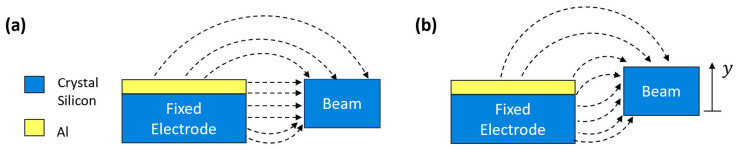
Electrostatic field of the actuator at (**a**) equilibrium (**b**) out-of-plane motion.

To account for the fringing field, we modify the capacitance expression on the right-hand side by multiplying it with a constant term denoted as β [31]

(2) $$\beta =\kappa (1.13\times 10^{-6}{\left(\frac{b}{d}\right)}^{3}-0.00019{\left(\frac{b}{d}\right)}^{2}+0.013\left(\frac{b}{d}\right)+1.638)$$

and κ is a factor that accounts for the geometry of the resonator’s fringing field during the out-of-plane motions; see Figure 3.

The governing equation of the electrical subsystem can be written as

(3) $$\ddot{q}+\frac{R}{L}\;\dot{q}+\frac{d}{L(C_{p}d+\epsilon hl+\epsilon |w_{\circ }-w|l)}\;q=\frac{V_{s}\left(t\right)}{L}$$

where *q* is the total charge stored on the parallel-plate actuator and $V_{s}\left(t\right)$ is the source voltage of the circuit.

#### Nondimensionalization

We introduced the following nondimensional parameters to nondimensionalize the equations of motion

(4) $$\hat{t}=\frac{t}{T}\;,\;\hat{w}=\frac{w}{\ell }\;,\;\hat{q}=\frac{q}{C_{\circ }}$$

where $T=\sqrt{\frac{m}{k}}$ is a time-scale, ℓ=0.01h is the resonator’s characteristic length and $C_{\circ }$ is the nominal capacitance at zero displacement and 1 V. Using Equation (4) in Equation (1) and multiplying by $\frac{T^{2}}{h}$, we obtain

(5) $$\ddot{\hat{w}}+\frac{1}{Q_{m}}\;\dot{\hat{w}}+\hat{w}=\frac{\epsilon \beta lT^{2}}{2\ell md}\;V{\left(\hat{t}\right)}^{2}\mathrm{sgn}\left({\hat{w}}_{\circ }-\hat{w}\right)$$

where the nondimensional natural frequency of the mechanical subsystem is set to unity, ${\hat{\omega }}_{m}=1$. Dispensing with the over-hat for the sake of simplicity, we can rewrite the equation of motion as

(6) $$\ddot{w}+\frac{1}{Q_{m}}\;\dot{w}+w=\alpha \;V{\left(t\right)}^{2}\;\mathrm{sgn}\left(w_{\circ }-w\right)$$

where a transduction parameter $\alpha =\;\frac{\epsilon \beta lT^{2}}{2\ell md}$ has been introduced to serve as the electromechanical coupling coefficient.

Substituting with Equation (4) into Equation (3), we obtain

(7) $$\frac{C_{\circ }}{T^{2}}\;\ddot{\hat{q}}+\frac{C_{\circ }}{T}\;\frac{R}{L}\;\dot{\hat{q}}+\frac{C_{\circ }\;d}{L\gamma (\hat{w})}\;\hat{q}=\frac{V_{s}\left(\hat{t}\right)}{L}$$

where the capacitance parameter can be written as

$$\gamma \left(\hat{w}\right)=C_{p}d+\ell \epsilon l+\ell \epsilon |{\hat{w}}_{\circ }-\hat{w}|l$$

Multiplying by $\frac{T^{2}}{C_{\circ }}$, and dropping the over-hat for the sake of simplicity, we can rearrange the nondimensional equation as

(8) $$\ddot{q}+\frac{\omega_{e}}{Q_{e}}\;\dot{q}+\omega_{e}^{2}q=\frac{T^{2}}{C_{\circ }}\;\frac{V_{s}\left(t\right)}{L}$$

where the electrical subsystem quality factor is defined as

$$Q_{e}=\frac{1}{R}\sqrt{\frac{Ld}{\gamma (w)}}$$

and a nondimensional parameter defined as

$$\omega_{e}=T\sqrt{\frac{d}{L\gamma (w)}}$$

has been introduced to describe the electrical natural frequency.

## 3. Results

### 3.1. Characterization

We characterized two electrostatically actuated MEMS resonators, dubbed actuator I and actuator II, as well as the circuits used to drive them under identical ambient air conditions. Then, we investigated their frequency response in primary resonance of the first out-of-plane bending mode under various actuation techniques. The actuators share the same design, as shown in Figure 1, and dimensions, as shown in Table 1.

Modal analysis was carried out to determine the lowest resonant frequencies of the actuators. The chip containing the actuator was placed into a DIP-40 chip carrier, and the actuators being tested were wire-bonded to it to avoid the parasitic incurred through probing.

**Figure 4:**
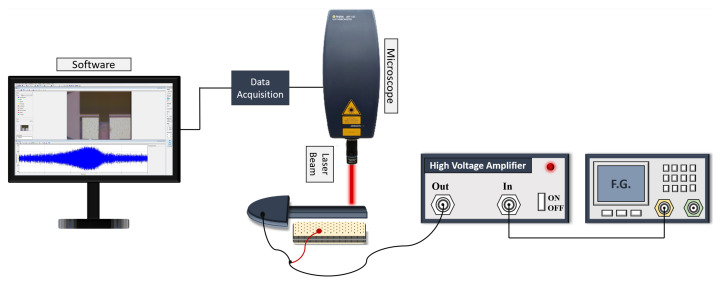
The experimental setup for actuator characterization.

The experimental setup shown in Figure 4 was utilized to characterize the actuators. The chip carrier is placed on a breadboard under the Polytec UHF-120 Laser Doppler Vibrometer (LDV) [32] to measure the beam tip displacement optically. The Liquid Instruments’ Moku:Pro [33] served as a function generator to feed the excitation signal to a high voltage amplifier, Tabor Electronics-9400 [34]. The actuation signal was supplied to the fixed electrode while the microbeam was grounded.

### 3.2. Modal Analysis

For the initial characterization, the damped natural frequencies of the actuators were obtained by applying a pulse train with a 0.01% duty cycle and a 312.5 Hz frequency to the fixed electrode. The Fast Fourier Transform (FFT) of the measured tip displacement to a single pulse was obtained using the LDV software, PSV [35], and averaged over 30 samples.

**Figure 5:**
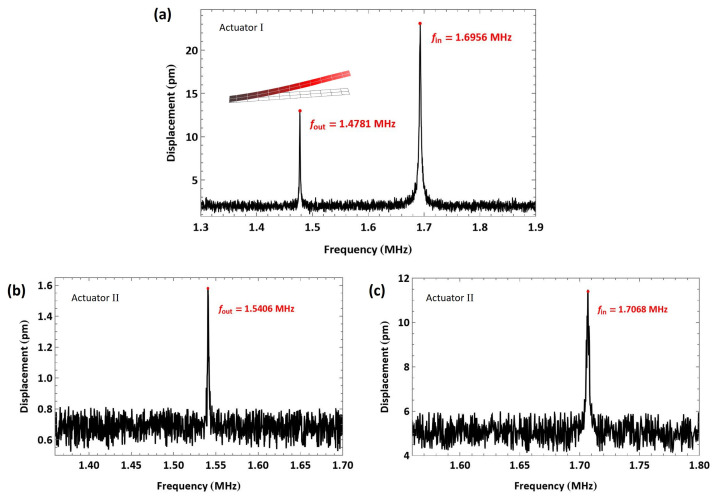
FFT of the measured tip displacements under pulse excitation, showing (**a**) the first out-of-plane and in-plane bending modes of actuator I, (**b**) the first out-of-plane, and (**c**) the first in-plane bending modes of actuator II.

For actuator I, the first out-of-plane bending mode was found at $f_{\mathrm{out}}=1.4781$ MHz, while the first in-plane bending mode was found at $f_{\mathrm{in}}=1.6956$ MHz; see Figure 5a. They were distinguished by the larger peak response of the in-plane mode, strongly actuated via the parallel part of the electrostatic field, compared to the smaller peak of the out-of-plane mode, weakly actuated via the fringing part of the electrostatic field. The quality factors of the modes were calculated using the half-power bandwidth method from the measured FFT as $Q_{\mathrm{out}}=1211$, corresponding to a settling time of $t_{s}=Q_{\mathrm{out}}T_{\mathrm{out}}=0.81929$ ms, and $Q_{\mathrm{in}}=720.5$, corresponding to a settling time of $t_{s}=Q_{\mathrm{in}}T_{\mathrm{in}}=0.42492$ ms.

For actuator II, the first out-of-plane bending mode was observed at $f_{\mathrm{out}}=1.5406$ MHz under a voltage amplitude of 100 V; see Figure 5b. The first in-plane bending mode was measured at $f_{\mathrm{in}}=1.7068$ MHz under an amplitude of 150 V; see Figure 5c. The quality factors of the modes were calculated using the half-power bandwidth method from the measured FFT as $Q_{\mathrm{out}}=1100.5$, corresponding to a settling time of $t_{s}=Q_{\mathrm{out}}T_{\mathrm{out}}=0.71432$ ms, and $Q_{\mathrm{in}}=685.5$, corresponding to a settling time of $t_{s}=Q_{\mathrm{in}}T_{\mathrm{in}}=0.40161$ ms.

Although both actuators share the same design, they exhibit different resonance frequencies and quality factors due to chip-to-chip variability in the fabrication process.

### 3.3. Conventional Excitation

We obtained the frequency response of the actuators under various excitation levels through a conventional excitation scheme following the experimental setup shown in Figure 4. These curves serve as the ‘ground truth’ for comparison with the results obtained from the resonant drive techniques.

The response of the first out-of-plane bending mode was measured under an unbiased voltage waveform. Due to the quadratic nature of the electrostatic force, applying a signal with a frequency *f* to the actuator results in an electrostatic excitation at 2f

(9) $$\begin{array}{c} F_{es}\left(t\right)\propto \alpha V{\left(t\right)}^{2}=\alpha {\left(V_{\mathrm{AC}}\cos\left(2\pi ft\right)\right)}^{2}=\frac{1}{2}\alpha V_{\mathrm{AC}}^{2}+\frac{1}{2}\alpha V_{\mathrm{AC}}^{2}\cos\left(4\pi ft\right) \end{array}$$

**Figure 6:**
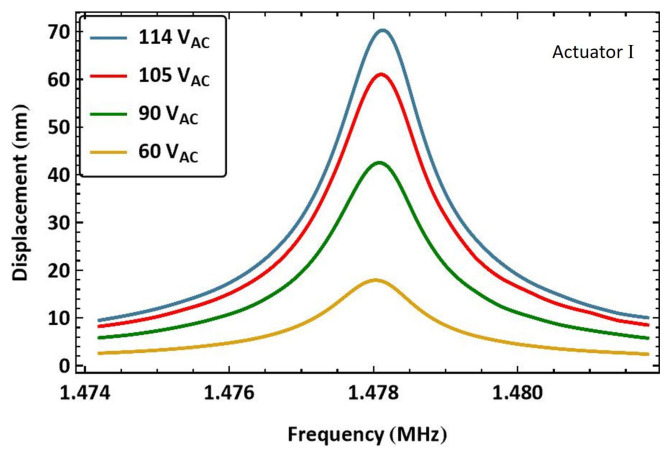
The frequency–response curves of actuator I beam tip displacement under four levels of the amplified voltage amplitude $V_{\mathrm{AC}}$.

For example, to excite actuator I, the signal frequency was swept up in the frequency range f=[737−741] kHz, resulting in an excitation frequency range of 2f=[1.474−1.482] MHz, which covers the half-power bandwidth of its first out-of-plane mode. The beam’s tip displacement was recorded over a time window of 100 ms, with a sampling frequency of 100 MHz. The sweep time was set to more than 10 times longer than the settling time $t_{s}$ to minimize transients in the response. The frequency–response curves, shown in Figure 6, were obtained for four signal amplitudes $V_{\mathrm{AC}}$ by post-processing the measured displacement to evaluate its RMS over a moving time window extending over 180 excitation periods [36]. The reported voltage amplitudes $V_{\mathrm{AC}}$ are those obtained from the voltage amplifier.

The largest RMS displacement for this excitation scheme was $w_{\mathrm{RMS}}=70.28$ nm obtained for the highest voltage amplitude $V_{\mathrm{AC}}=114$ V at resonance. It corresponds to only 3.5% of the air gap. It is worth mentioning that the voltage amplifier utilized to obtain those curves was operating beyond its specifications. Although its declared gain is 50×, the input voltage to the MEMS measured by an oscilloscope was found to have a gain of 60× in this frequency range. This is characteristic behavior of a low-pass filter, such as a voltage amplifier, where the gain may temporarily increase when operating near its resonance (cutoff) frequency [37].

**Figure 7:**
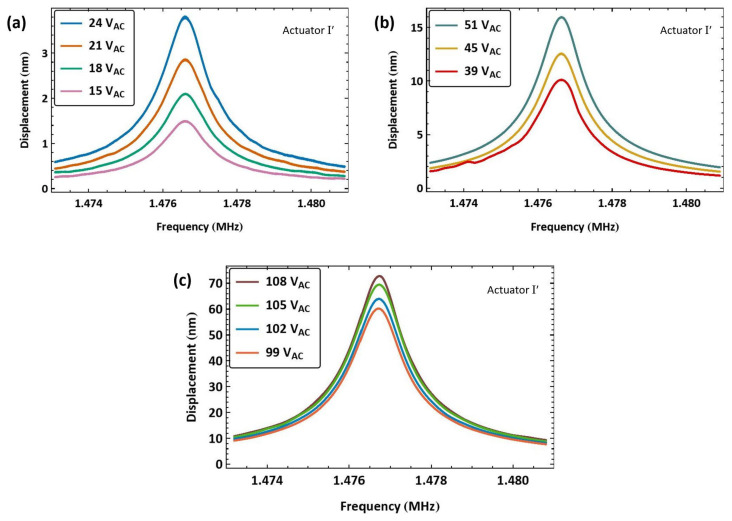
The frequency-response curves of the beam tip displacement of actuator $I^{\prime }$ under eleven levels of the amplified voltage amplitude $V_{\mathrm{AC}}$ (**a**–**c**).

During the experimental campaign, it was observed that the resonant frequency of actuator I dropped by 500 Hz, indicating a degradation in its stiffness. Results obtained after degradation are dubbed as those of actuator $I^{\prime }$. Figure 7 shows the frequency response of actuator $I^{\prime }$ to eleven unbiased and amplified voltage amplitudes $V_{\mathrm{AC}}$. For comparison, under a voltage amplitude of $V_{\mathrm{AC}}=105$ V, the peak response of actuator I, shown in Figure 6, is $w_{\mathrm{RMS}}=61$ nm, whereas that of actuator $I^{\prime }$ is $w_{\mathrm{RMS}}=69$ nm, shown in Figure 7c, indicating a drop in beam stiffness.

**Figure 8:**
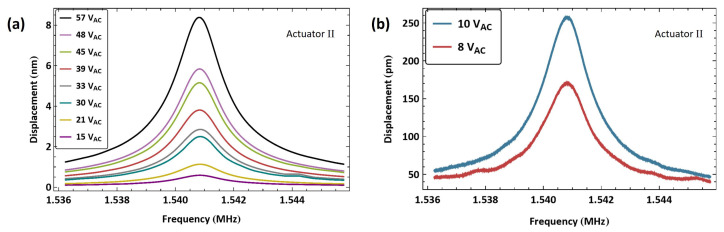
The frequency–response curves of actuator II beam tip displacement for (**a**) eight levels of the amplified voltage amplitude $V_{\mathrm{AC}}$ and (**b**) two levels of the voltage amplitude $V_{\mathrm{AC}}$.

The frequency response of actuator II was obtained using the same experimental procedure. The frequency–response curves for eight levels of the amplified voltage amplitude $V_{\mathrm{AC}}$ are shown in Figure 8a. The excitation frequency was swept up in the vicinity of the first out-of-plane bending mode over a frequency range of 2f=[1.536−1.546] MHz. The maximum measured displacement was $w_{\mathrm{RMS}}=8.3$ nm, observed at resonance under a voltage amplitude of $V_{\mathrm{AC}}=57$ V.

For comparison, we show in Figure 8b the frequency response of actuator II driven directly by the function generator alone for two signal amplitudes. We note that the maximum RMS displacement achieved was $w_{\mathrm{RMS}}=250$ pm, which is only 0.01285% of the air gap.

### 3.4. Resonant Drive

We describe the experimental procedure used to characterize the circuits of the resonant drive techniques, the characterization of those circuits, and the measured response of the actuators under these excitation regimes.

#### 3.4.1. Electrical Characterization

**Figure 9:**
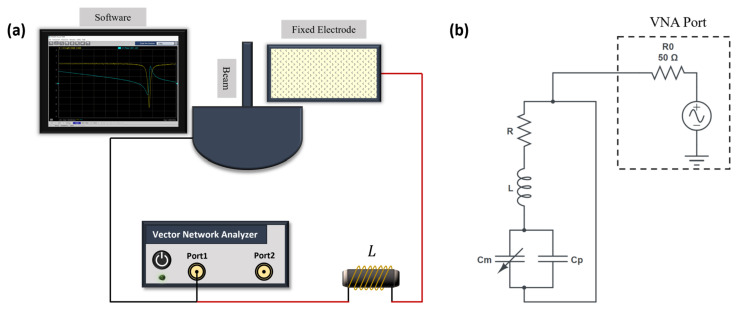
(**a**) The experimental setup for resonant drive circuit characterization. (**b**) The equivalent circuit.

The experimental setup shown in Figure 9a was used to measure the natural frequency $f_{e}$ of series RLC circuits consisting of an external inductor *L*, the circuit parasitic resistance *R*, the capacitance of the actuator $C_{m}$, and the circuit parasitic capacitance $C_{p}$. A lumped-element representation of the circuit is shown in Figure 9b. The measurements were carried out using a Vector Network Analyzer (VNA) (Keysight P5020A). To simplify the measurement process, only one-port S-parameter $S_{11}$ measurements were undertaken. This represents the ratio of the reflected wave to the incident wave as a function of frequency at the input port of the circuit. A calibration process was performed prior to measurements to account for the parastics presented by cables and connectors. Calibration was carried out using the short-open-load calibration approach with the Keysight 85521A Mechanical Calibration Kit.

Different inductors were used with the resonant circuit to achieve various resonance frequencies required for the resonant drive techniques discussed in the following sections. Below, the characterization of each inductor is detailed.

**Figure 10:**
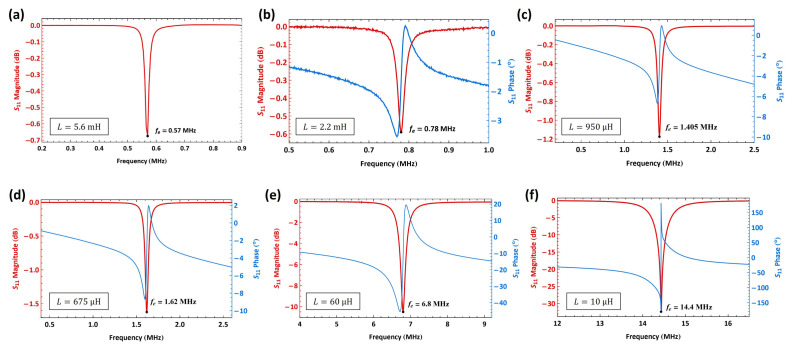
The measured $S_{11}$ parameter of an RLC circuit composed of a MEMS actuator and an external inductor where (**a**) L=5.6 mH; (**b**) L=2.2 mH; (**c**) L=950 µH; (**d**) L=675 µH; (**e**) L=60 µH; and (**f**) L=10 µH.

For the characterization with a 5.6 mH inductor, composed of two series-connected inductors (2.4 mH and 3.2 mH), the power level of the VNA was set to 0 dBm (1 mW). The input signal frequency was swept over the range f=[0.15−15] MHz with a step frequency of 5 kHz. The intermediate frequency bandwidth (IF-BW) was set to 1 kHz. The amplitude of the $S_{11}$ response of the RLC circuit, shown in Figure 10a in logarithmic (dB) scale, indicates that electrical resonance occurred at $f_{e}=0.57$ MHz, corresponding to the minimum value of $S_{11}=-0.674$ dB. The electrical quality factor of the RLC circuit was calculated using the half-power bandwidth method from the measured $S_{11}$ magnitude, where the bandwidth was determined from a linear scale (rather than dB), yielding $Q_{e}=38$. It was also found that the natural frequencies of the circuits were insensitive to the MEMS actuator in them but varied with the inductor and parasitics in the circuit.

The characterization procedure was repeated for an RLC circuit with a 2.2 mH connected in series with the actuator, Figure 10b. The signal frequency was swept over f=[0.1−1] MHz, with a step frequency of 1 kHz. The IF-BW was set to 1 kHz, and the data were averaged over five samples. The amplitude of $S_{11}$ is represented in a dB scale, while the phase is shown in degrees. The electrical resonance was found at the minimum value of the $S_{11}=-0.590$ dB as $f_{e}=780$ kHz. The corresponding phase angle swung from a minimum of $-3.5^{\circ }$ to a maximum of $0.25^{\circ }$. The quality factor of the circuit was calculated as $Q_{e}=60$.

The 950 µH and 675 µH inductors were characterized using the same settings as the 5.6 mH inductor, including the signal frequency sweep range, step size, and IF-BW. Figure 10c shows the $S_{11}$ amplitude and phase response when a 950 µH inductor was connected to the actuator. The electrical resonance was observed at $f_{e}=1.405$ MHz at $S_{11}=-1.17$ dB. At resonance, the phase angle varied from $-6.67^{\circ }$ to $0.94^{\circ }$. The quality factor was determined to be $Q_{e}=46$.

For the 675 µH inductor, the $S_{11}$ amplitude and phase response are shown in Figure 10d. The electrical resonance occurred at a minimum value of $S_{11}=-1.62$ dB corresponding to a frequency of $f_{e}=1.62$ MHz. The phase angle at resonance ranged from $-8.68^{\circ }$ to $1.96^{\circ }$ , and the quality factor was calculated as $Q_{e}=36$.

Next, we characterized the resonant circuit with 60 µH and 10 µH inductors. The signal frequency was swept in the range f=[3−15] MHz and the IF-BW was set to 2 kHz. The amplitude and phase of the frequency response of the RLC circuit are shown in Figure 10e when a 60 µH was connected to the actuator. The electrical resonance was found at the minimum value of $S_{11}=-10.467$ dB to be $f_{e}=6.8$ MHz, and the corresponding phase angle swung from a minimum of $-45.4^{\circ }$ to a maximum of $19.4^{\circ }$. The calculated quality factor in this case was found to be $Q_{e}=48$.

Figure 10f shows the $S_{11}$ magnitude and phase of the frequency response of the resonant circuit when a 10 µH inductor is connected with the actuator. The electrical resonance was found at the minimum value of $S_{11}=-32.073$ dB to be $f_{e}=14.4$ MHz and the corresponding phase angle swung from a minimum of $-176.6^{\circ }$ to a maximum of $178.3^{\circ }$. The calculated quality factor in this case was found to be $Q_{e}=65$.

#### 3.4.2. Experimental Setup

**Figure 11:**
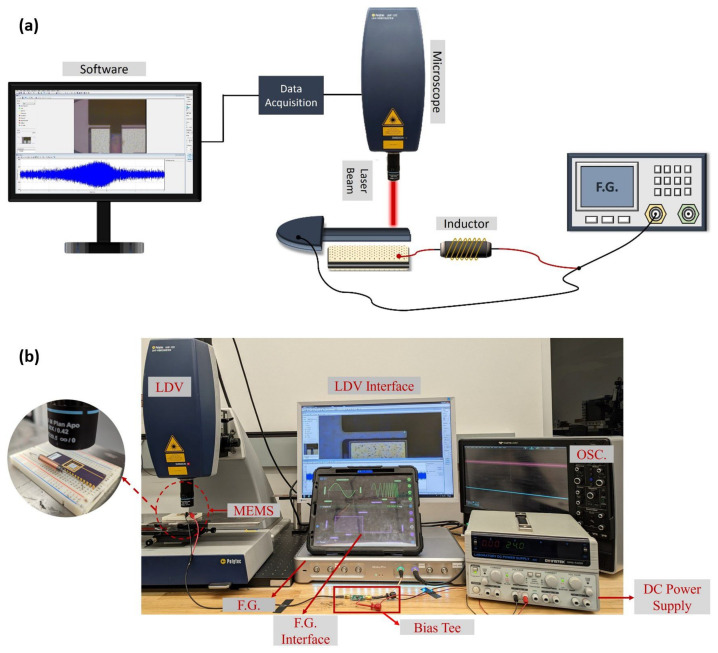
(**a**) A schematic and (**b**) a picture of the experimental setups of unbiased and biased resonant drive experiments, respectively.

The experimental setup for the resonant drive experiments is shown in Figure 11. The fixed electrode of the actuator is connected in series to an external inductor to tune the electrical natural frequency of the circuit. The LDV [32] is used to measure the tip displacement. The ground of the function generator (Liquid Instruments’ Moku:Pro [33]) is connected to the beam while it supplies the drive signal of the circuit. Voltage amplification is achieved by the dynamic amplification $\left(Q_{e}\right)$ of the RLC circuit.

Figure 11a presents the experimental setup used to excite the actuator with unbiased signals, whereas Figure 11b depicts the setup used for biased signals. In the latter case, a bias tee and a DC power supply are introduced to the circuit.

#### 3.4.3. Resonance Matching

The first technique employed was resonance matching. It calls for the use of a single tone signal designed to match the electrical resonance $f_{e}$ of the RLC circuit and to result in an electrostatic excitation force with a dominant peak that matches the mechanical resonance $f_{m}$ of the actuator. For unbiased signals, the external inductor *L* is sized such that $f_{e}\approx \frac{1}{2}f_{m}$ as per Equation (9). For biased signals, the electrostatic force is described by

(10) $$\begin{array}{cc} F_{es}\left(t\right)\propto \alpha V{\left(t\right)}^{2} & =\alpha {\left(V_{\mathrm{DC}}+V_{\mathrm{AC}}\cos\left(2\pi ft\right)\right)}^{2}\\ \; & =\alpha V_{\mathrm{DC}}^{2}+\frac{1}{2}\alpha V_{\mathrm{AC}}^{2}+2\alpha V_{\mathrm{DC}}V_{\mathrm{AC}}\cos\left(2\pi ft\right)+\frac{1}{2}\alpha V_{\mathrm{AC}}^{2}\cos\left(4\pi ft\right) \end{array}$$

and the dominant peak is at $f_{e}$. Therefore, the external inductor *L* is sized such that $f_{e}\approx f_{m}$.

To excite actuator I with an unbiased signal, an L=2.2 mH inductor was connected in series with the actuator, resulting in a VNA-measured electrical resonance of $f_{e}=780$ kHz, Figure 10b. It was found that the electrical natural frequency drops in the drive setup, shown in Figure 11a, due to uncharacterized reactive loading introduced by the function generator into the circuit. Hence, we intentionally set the electrical natural frequency to a value larger than the desired signal frequency $f_{e}>f$. The signal frequency was swept within the range f=[737−741] kHz in a time window of 100 ms while the voltage amplitude $V_{\mathrm{AC}}$ was held constant.

**Figure 12:**
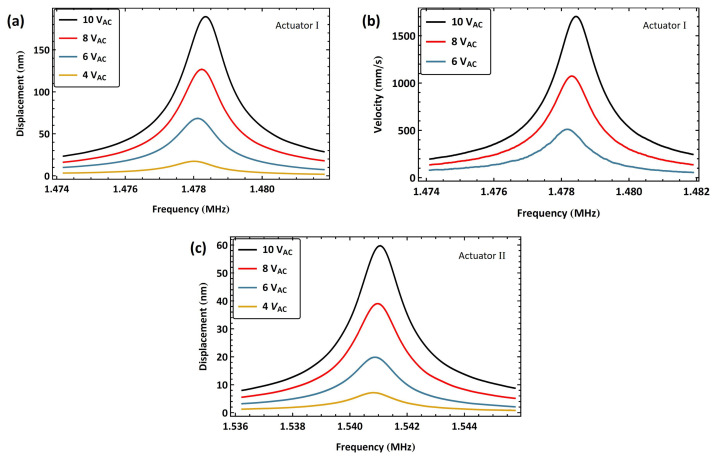
The frequency–response curves of the beam tip for two actuators under resonance matching excitation with a 2.2 mH inductor. (**a**) Displacement of actuator I at four unbiased voltage amplitudes, $V_{\mathrm{AC}}$. (**b**) Velocity of actuator I at three unbiased voltage amplitudes $V_{\mathrm{AC}}$. (**c**) Displacement of actuator II at four unbiased voltage amplitudes $V_{\mathrm{AC}}$.

The frequency response of actuator I is presented in Figure 12a for four values of the voltage amplitude $V_{\mathrm{AC}}$. For a voltage amplitude of $V_{\mathrm{AC}}=10$ V, this excitation scheme achieves a maximum displacement of $w_{\mathrm{RMS}}=189$ nm at resonance, which is more than twice the maximum displacement attained using a conventional voltage amplifier, Figure 6. The peak of the frequency–response curves was observed to shift to the right. Therefore, the experiment was repeated using a longer time window to investigate whether this shift was due to the hardening of the resonator or an artifact of the sweep transients. The tip velocity of actuator I was measured using the OFV-5000 LDV [38], and the data were collected by a digital oscilloscope in 4 s long time windows. The actuator velocity, Figure 12b, demonstrates a similar hardening behavior as the voltage amplitude is increased from $V_{\mathrm{AC}}=6$ V to 10 V, reaching a peak of $V_{\mathrm{RMS}}=1.703$ m/s, which confirms the presence of a hardening nonlinearity. Given the relatively small displacements involved, the source of this nonlinearity is probably the electrostatic field rather than the mechanical potential.

The VNA-measured electrical resonance was unchanged at $f_{e}=780$ kHz once the chip carrying actuator II was introduced into the circuit. To excite primary resonance, the signal frequency was swept within the range f=[768−773] kHz in a time window of 100 ms. Figure 12c shows the frequency response of actuator II tip displacement for four values of the voltage amplitude $V_{\mathrm{AC}}$. The largest realized displacement for $V_{\mathrm{AC}}=10$ V was $w_{\mathrm{RMS}}=59.7$ nm, corresponding to 2.99% of the air gap. The lower peak amplitude in this case is due to a lower quality factor and higher stiffness than those of actuator I. Similar to actuator I, the peak of the frequency response curves was observed to shift to the right, confirming the presence of a hardening nonlinearity.

**Figure 13:**
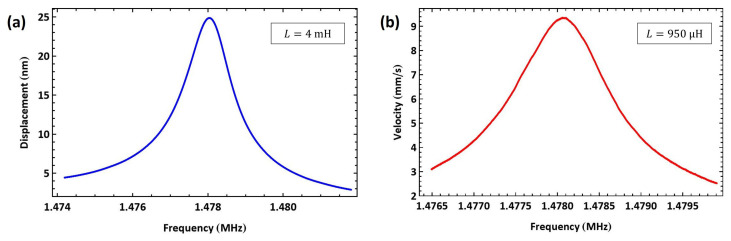
The frequency–response curves of actuator I under mistuned resonance matching excitation. (**a**) Tip displacement with an oversized inductor (L=4 mH). (**b**) Velocity with an undersized inductor (L=950 µH).

We deliberately oversized the external inductor to L=4 mH to investigate the impact of mistuning the electrical and mechanical resonances away from $f_{e}\approx \frac{1}{2}f_{m}$ on our actuation technique. The VNA-measured electrical resonance was found to be $f_{e}=593$ kHz, well below half of the mechanical resonance of actuator I at $\frac{1}{2}f_{m}=739$ kHz. Figure 13a shows the frequency–response curve of actuator I tip displacement under this excitation regime and a voltage amplitude of $V_{\mathrm{AC}}=10$ V as the signal frequency was swept from f=737 kHz to 741 kHz over a time window of 100 ms. The actuator displacement dropped, as shown in Figure 13a, by one order of magnitude to $w_{\mathrm{RMS}}=25$ nm, which is much lower than the displacement obtained in the tuned case.

Likewise, we undersized the external inductor to L=950 µH, resulting in a VNA-measured electrical resonance of $f_{e}=1.405$ MHz, as shown in Figure 10c, well above half of the mechanical resonance of actuator I. Figure 13b shows the frequency–response curve of actuator I tip velocity under a voltage amplitude of $V_{\mathrm{AC}}=10$ V as the signal frequency swept in the range of f=[738.2−740] kHz over a time window of 30 ms. The tip velocity dropped by two orders of magnitude to $V_{\mathrm{RMS}}=9$ mm/s.

**Figure 14:**
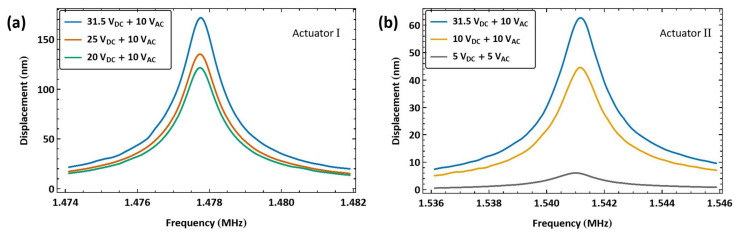
The frequency–response curves of the beam tip displacement for (**a**) actuator I under resonance matching excitation at three bias levels and a voltage amplitude of $V_{\mathrm{AC}}=10$ V. The actuator is connected in series with two inductors: $L_{1}=470$ µH and $L_{2}=27$ µH. (**b**) actuator II under resonance matching excitation at three bias levels for two voltage amplitudes, $V_{\mathrm{AC}}=10$ V and $V_{\mathrm{AC}}=5$ V. The actuator is connected in series with an inductor L=675 µH inductor.

The experimental setup shown in Figure 11b was adopted to implement biased resonance matching excitation. In this setup, a DC power supply and a bias Tee are introduced to add a bias to the harmonic signal generated by the function generator. For actuator I, two external inductors, $L_{1}=470$ µH and $L_{2}=27$ µH, were connected in series to match the electric and mechanical natural frequencies $f_{e}\approx f_{m}$. The signal frequency was swept over the range f=[1.474−1.482] MHz. Figure 14a shows the frequency response of the actuator under three levels of bias voltage $V_{\mathrm{DC}}$ and a voltage amplitude of $V_{\mathrm{AC}}=10$ V. The maximum measured displacement $w_{\mathrm{RMS}}=171.6$ nm was realized at resonance for a bias voltage of $V_{\mathrm{DC}}=31.5$ V.

We note that the introduction of bias into the signal degrades the efficiency of the resonance-matching technique. This can be seen in the fact that adding a bias voltage of $V_{\mathrm{DC}}=31.5$ V to the same harmonic signal ($V_{\mathrm{AC}}=10$ V) ends up reducing the realized displacement, in addition to incurring the cost and complexity of biasing the signal and operating in a higher frequency range.

Similarly, we explored the response of actuator II under biased resonance matching. The signal frequency was swept across the range f=[1.536−1.546] MHz while connecting an external inductor L=675 µH, resulting in a VNA-measured electrical resonance of $f_{e}=1.62$ MHz, as shown in Figure 10d. Figure 14b shows the frequency response curves of actuator II under three bias voltage levels $V_{\mathrm{DC}}$ and two voltage amplitudes $V_{\mathrm{AC}}$. The discrepancy in the external inductors required for resonance matching between actuators I and II appears to be a result of unaccounted-for parasitic inductance in the case of actuator I.

The largest achieved displacement for actuator II was $w_{\mathrm{RMS}}=62.67$ nm for a bias voltage of $V_{\mathrm{DC}}=31.5$ V and a voltage amplitude of $V_{\mathrm{AC}}=10$ V, which is marginally larger than that obtained with the same unbiased signal ($V_{\mathrm{AC}}=10$ V). This result confirms our conclusion about the effectiveness of the unbiased compared to the biased technique.

**Figure 15:**
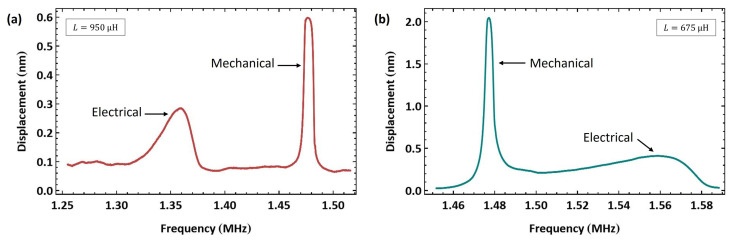
The frequency–response curve of actuator $I^{\prime }$ tip displacement under a mistuned resonance matching excitation with (**a**) an oversized inductor (L=950 µH) and (**b**) an undersized inductor (L=675 µH).

We investigated the impact of mistuning on resonance matching with biased signals. Due to the limited drive voltage used in this experiment, a bias Tee was not required, and the experimental setup of Figure 11a was adopted. First, a significantly oversized external inductor with L=950 µH was introduced to the drive circuit. The VNA-measured electrical resonance was $f_{e}=1.405$ MHz, as shown in Figure 10c, mistuning the electrical resonance to the lower side of the mechanical resonance $f_{e}<f_{m}$. Figure 15a shows the measured frequency–response curve of actuator $I^{\prime }$ tip displacement under resonance matching excitation with the voltage waveform $V_{\mathrm{AC}}=V_{\mathrm{DC}}=5$ V as the signal was swept in the frequency range f=[1.25−1.52] MHz over a time window of 200 ms. The electrical and mechanical resonances are mismatched, with the former appearing at $f_{e}=1.359$ MHz and the latter appearing at $f_{m}=1.477$ MHz.

Since the resonance frequency of actuator I’ is lower than that of actuator II, we used the inductor of the latter L=675 µH as an undersized inductor for the former. Figure 15b shows the measured frequency–response curve of actuator $I^{\prime }$ tip displacement under the same voltage waveform ($V_{\mathrm{AC}}=V_{\mathrm{DC}}=5$ V) as the signal was swept in the frequency range f=[1.45−1.59] MHz over a time window of 100 ms. Distinct peaks corresponding to the mismatched electrical and mechanical resonances appear in the frequency response with the electrical resonance at $f_{e}=1.56$ MHz and the mechanical resonance at $f_{m}=1.477$ MHz. Note that the VNA-measured electrical resonance was $f_{e}=1.62$ MHz, as shown in Figure 10d.

The previous cases demonstrate that optical measurement of the MEMS response can serve as an indicator of the precise location of electrical resonance $f_{e}$ in the actuation circuit. To accurately match the two resonances $\left(f_{e}=f_{m}\right)$, the electrical resonance $f_{e}$ should be observed optically under a biased signal and adjusted iteratively until it merges with the mechanical resonance $f_{m}$ into a single peak. Note that when this technique is used to tune the two resonances for an unbiased signal, the two peaks observed in the same vicinity are the electrical resonance $f_{e}$ and the superharmonic $\frac{1}{2}f_{m}$, rather than primary mechanical resonance.

#### 3.4.4. Multi-Frequency Excitation

The multi-frequency excitation technique utilizes the summation of two voltage signals, resulting in an electrostatic force of the form

(11) $$\begin{array}{cc} F_{es}\left(t\right)\propto \alpha V{\left(t\right)}^{2} & =\alpha {\left(V_{\mathrm{AC}1}\cos\left(2\pi f_{1}t\right)+V_{\mathrm{AC}2}\cos\left(2\pi f_{2}t\right)\right)}^{2}\\ & =\frac{\alpha }{2}\left(V_{\mathrm{AC}1}^{2}+V_{\mathrm{AC}2}^{2}\right)+\frac{\alpha }{2}\left(V_{\mathrm{AC}1}^{2}\cos\left(4\pi f_{1}t\right)+V_{\mathrm{AC}2}^{2}\cos\left(4\pi f_{2}t\right)\right)\\ & \;+\alpha V_{\mathrm{AC}1}V_{\mathrm{AC}2}\left(\cos\left(2\pi (f_{1}-f_{2})t\right)+\cos\left(2\pi (f_{1}+f_{2})t\right)\right) \end{array}$$

**Figure 16:**
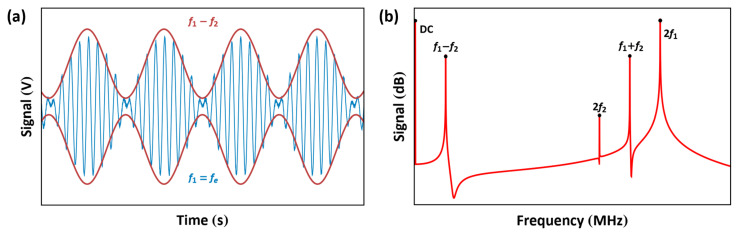
Representations of the multi-frequency signal in (**a**) the time and (**b**) frequency domains.

The frequency of one signal is set to be equal to the electric resonance of the RLC circuit $f_{1}\approx f_{e}$ while the sum or difference of the frequencies of the two signals is set equal to the mechanical resonance $f_{1}\pm f_{2}=f_{m}$ to drive the actuator with one of the last terms on the right-hand side of Equation (11). Figure 16 shows the signal for the case of $f_{1}>f_{2}$.

As noted in Section 3.4.3, it was found that the reactive loading of the function generator in the drive setup, shown in Figure 11a, causes a drop in the electrical resonance away from that measured using the VNA. To address this, a two-step process was developed to determine the electrical resonance $f_{e}$. First, an estimate of the electrical resonance ${\hat{f}}_{e}$ was obtained using the VNA and the setup of Figure 9a to measure the electrical resonance. Then, the drive setup, shown in Figure 11a, was used to vary the first signal frequency $f_{1}$ in the vicinity of ${\hat{f}}_{e}$ while measuring the response of the actuator. The electrical resonance $f_{e}$ was determined as the first signal frequency $f_{1}$ at the peak response.

First, we tested the use of a higher electric resonance $\left(f_{e}>f_{m}\right)$ in multi-frequency excitation, which corresponds to driving the actuator via the electrostatic force of the frequency difference $\left(f_{1}-f_{2}\right)$. Actuator $I^{\prime }$ was connected in series to an external inductor $L_{1}=60$ µH resulting in an estimated electrical resonance of ${\hat{f}}_{e}=6.8$ MHz; see Figure 10e. Based on that, the electrical resonance was found to be $f_{e}=6.27$ MHz using the search routine described above. The frequency of the first signal was set to match the electrical resonance frequency $f_{1}=f_{e}$. The frequency of the second signal was swept down in the range $f_{2}=\left[4.797-4.789\right]$ MHz over 100 ms, so that the difference between the two frequencies was swept up in the vicinity of the mechanical natural frequency $f_{1}-f_{2}=\left[1.473-1.481\right]$ MHz.

**Figure 17:**
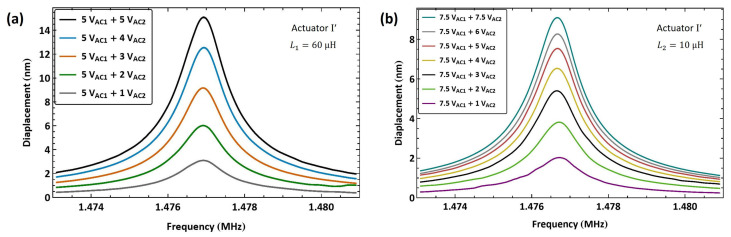
The frequency response of actuator $I^{\prime }$ tip displacement under multi-frequency excitation. (**a**) $f_{e}=6.27$ MHz. (**b**) $f_{e}=13.25$ MHz.

Figure 17a shows the frequency–response curves of actuator $I^{\prime }$ tip displacement under five levels of the second signal voltage amplitude $V_{\mathrm{AC}2}$ while the amplitude of the amplifier (first) signal is held constant at $V_{\mathrm{AC}1}=5$ V. The maximum displacement realized was $w_{\mathrm{RMS}}=15$ nm, observed at resonance under a voltage amplitude of $V_{\mathrm{AC}2}=5$ V, which is 0.75% of the air gap. The response under this excitation scheme is well regulated as the second signal amplitude is varied.

To test the flexibility of this technique, we tuned the the electrical resonance to a higher value by using a smaller external inductor $L_{2}=10$ µH. As a result, the estimated electrical resonance increased to ${\hat{f}}_{e}=14.4$ MHz; see Figure 10f. The electrical resonance was found to be $f_{e}=13.25$ MHz using the drive setup. The first signal frequency was set equal to it $f_{1}=f_{e}$ , while the second signal frequency was swept down in the range $f_{2}=\left[11.777-11.769\right]$ MHz over 100 ms. Figure 17b shows the frequency response of actuator $I^{\prime }$ under a fixed amplifier voltage amplitude $V_{\mathrm{AC}1}=7.5$ V and seven levels of the second signal voltage amplitude $V_{\mathrm{AC}2}$. The maximum measured displacement with this inductor was $w_{\mathrm{RMS}}=9$ nm for a voltage amplitude of $V_{\mathrm{AC}2}=7.5$ V, corresponding to 0.45% of the air gap.

The drop in displacement with the increase in the amplifier signal voltage from $V_{\mathrm{AC}1}=5$ V to 7.5 V and the actuation signal voltage from $V_{\mathrm{AC}2}=5$ V to 7.5 V is an interesting finding. It is even more so given that the smaller inductor $L_{2}$ in the second case imposes lower losses on the RLC circuit due to a smaller equivalent series resistance (ESR) than the inductor $L_{1}$ of the first case. We postulate that the ESRs of both inductors are negligible, as they are in the µH range, compared to the overall resistance of the actuation circuit. On the other hand, the larger inductor $L_{1}$ results in a better electrical quality factor than the smaller inductor $L_{2}$ given that

$$Q_{e}=\frac{1}{R}\sqrt{\frac{L}{C}}$$

thereby making for a better amplifier circuit.

We applied the same excitation technique to actuator II to verify the scheme repeatability. An external inductor $L_{1}=60$ µH was connected in series with the actuator, resulting in an electrical resonance of $f_{e}=6.21$ MHz. The frequency of the amplifier signal was set to match the electrical resonance $f_{1}=f_{e}$. The frequency of the second signal was swept down in the range $f_{2}=\left[4.674-4.664\right]$ MHz over 100 ms so that the difference between the two frequencies was swept up in the vicinity of the mechanical natural frequency $f_{1}-f_{2}=\left[1.536-1.546\right]$ MHz.

**Figure 18:**
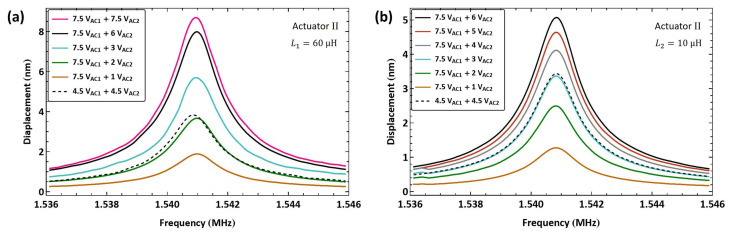
The frequency response of actuator II tip displacement under multi-frequency excitation. (**a**) $f_{e}=6.21$ MHz. (**b**) $f_{e}=13.5$ MHz.

Figure 18a shows the frequency response of actuator II under a fixed amplifier voltage amplitude $V_{\mathrm{AC}1}=7.5$ V and five levels of second signal voltage amplitude $V_{\mathrm{AC}2}$. The largest realized displacement with this inductor was $w_{\mathrm{RMS}}=8.7$ nm for a voltage amplitude of $V_{\mathrm{AC}2}=7.5$ V, corresponding to 0.435% of the air gap. For comparison, the figure also incorporates a frequency–response curve with a lower amplifier voltage amplitude of $V_{\mathrm{AC}1}=4.5$ V and an actuation voltage amplitude of $V_{\mathrm{AC}2}=4.5$ V. It can be seen that the response in the latter case is comparable to that of the higher amplifier voltage under a much smaller actuation voltage of $V_{\mathrm{AC}2}=2$ V, thereby demonstrating the efficacy of the amplifier circuit.

A smaller external inductor $L_{2}=10$ µH was also used in conjunction with actuator II, resulting in an electrical resonance frequency of $f_{e}=13.5$ MHz. The first signal frequency was set equal to it, $f_{1}=f_{e}$, while the second signal frequency was swept down in the range $f_{2}=\left[11.964-11.954\right]$ over 100 ms. Figure 18b shows the frequency response of actuator II tip displacement under seven levels of the second signal voltage amplitude while the amplitude of the first amplifier signal was held constant $V_{\mathrm{AC}1}=7.5$ V. The maximum measured tip displacement was $w_{\mathrm{RMS}}=5.1$ nm for a voltage amplitude of $V_{\mathrm{AC}2}=6$ V, corresponding to 0.255% of the air gap.

Similar to the case of actuator $I^{\prime }$, the realized tip displacement using the larger $L_{1}$ inductor was greater than that observed with the smaller $L_{2}$ inductor, thus confirming our earlier hypothesis. Figure 18b also incorporates a frequency–response curve with a lower amplifier voltage amplitude of $V_{\mathrm{AC}1}=4.5$ V and an actuation voltage amplitude of $V_{\mathrm{AC}2}=4.5$ V. It can be seen that the response in this case is comparable to that of the higher amplifier voltage under a smaller actuation voltage of $V_{\mathrm{AC}2}=3$ V, which confirms the efficacy of the amplifier circuit.

Next, we tested the use of a lower electric resonance $\left(f_{e}<f_{m}\right)$ to drive the actuator via the electrostatic force of the frequency sum $\left(f_{1}+f_{2}\right)$. Actuator $I^{\prime }$ was connected in series to a large inductor, made of two inductors $L_{3}=3.2$ mH and $L_{4}=2.4$ mH connected in series, resulting in an estimated electrical resonance of ${\hat{f}}_{e}=570$ kHz, Figure 10a. The electrical resonance was found to be $f_{e}=528$ kHz using the drive setup. The first signal frequency was set equal to the electrical resonance $f_{1}=f_{e}$ while the second signal frequency was swept up in the range $f_{2}=\left[945-953\right]$ kHz over 100 ms, so that the frequency sum was swept past the mechanical resonance $f_{m}$ in the range $f_{1}+f_{2}=\left[1.473-1.481\right]$ MHz.

**Figure 19:**
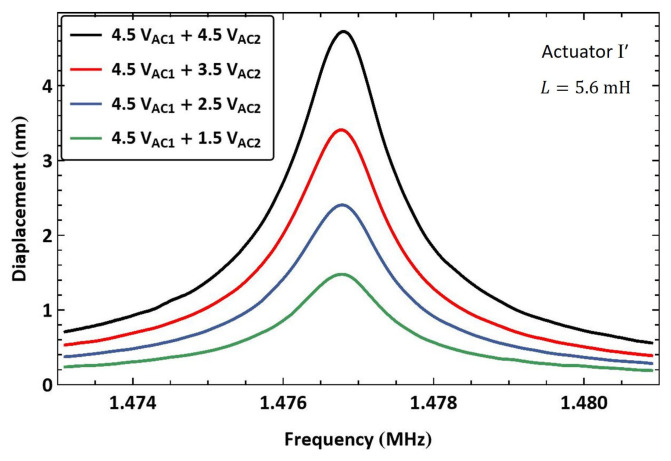
The frequency response of actuator $I^{\prime }$ tip displacement under multi-frequency excitation ($f_{e}=528$ kHz).

The frequency–response curves of actuator $I^{\prime }$ tip displacement are shown in Figure 19 for four values of the second signal voltage amplitude $V_{\mathrm{AC}2}$ while the amplifier voltage amplitude was held constant at $V_{\mathrm{AC}1}=4.5$ V. The largest displacement in this case was $w_{\mathrm{RMS}}=4.7$ nm, realized at resonance for a voltage amplitude of $V_{\mathrm{AC}2}=4.5$ V, corresponding to 0.235% of the air gap.

#### 3.4.5. Amplitude Modulation

In this excitation scheme, we utilize an amplitude-modulated signal, resulting in an electrostatic force of the form

(12) $$\begin{array}{cc} F_{es}\left(t\right)\propto \alpha V{\left(t\right)}^{2} & =\alpha {\left(V_{\mathrm{AC}1}+V_{\mathrm{AC}2}\cos\left(2\pi f_{b}t\right)\right)}^{2}\cos^{2}\left(2\pi f_{e}t\right)\\ & =\frac{\alpha }{4}\left(2V_{\mathrm{AC}1}^{2}+V_{\mathrm{AC}2}^{2}\right)+\frac{\alpha }{4}\left(2V_{\mathrm{AC}1}^{2}+V_{\mathrm{AC}2}^{2}\right)\cos\left(4\pi f_{e}t\right)\\ & \;+\frac{\alpha }{2}V_{\mathrm{AC}1}V_{\mathrm{AC}2}\left(\cos\left(2\pi (2f_{e}-f_{b})t\right)+\cos\left(2\pi (2f_{e}+f_{b})t\right)\right)\\ & \;+\frac{\alpha }{8}V_{\mathrm{AC}2}^{2}\left(\cos\left(4\pi (f_{e}-f_{b})t\right)+\cos\left(4\pi (f_{e}+f_{b})t\right)\right)\\ & \;+\alpha V_{\mathrm{AC}1}V_{\mathrm{AC}2}\cos\left(2\pi f_{b}t\right)+\frac{\alpha }{4}V_{\mathrm{AC}2}^{2}\cos\left(4\pi f_{b}t\right) \end{array}$$

**Figure 20:**
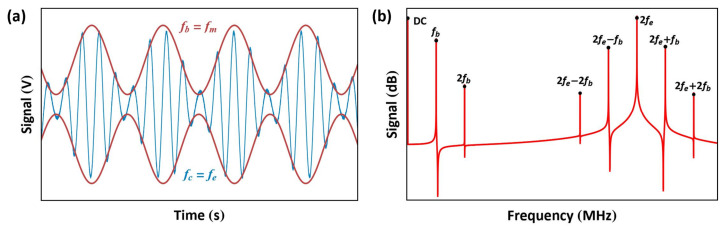
Representations of the amplitude-modulated signal in (**a**) the time and (**b**) frequency domains. $f_{e}$ is the carrier frequency and $f_{b}$ is the baseband frequency.

The drive signal, shown in Figure 20, is the product of an RF carrier signal with a frequency $f_{e}$ and a baseband signal with a frequency $f_{b}$. Given that $f_{e}$ is, by definition, much larger than $f_{b}$, the actuator acts as a low-pass filter, thereby attenuating the higher frequency forces at $2f_{e}$, $2f_{e}\pm f_{b}$, and $2f_{e}\pm 2f_{b}$. To guarantee this, the RF carrier frequency $f_{e}$ is typically set in the range of 3–100$f_{m}$[23]. On the other hand, the actuator follows the electrostatic force generated by the envelope of the signal. Provided that we set $V_{\mathrm{AC}1}>V_{\mathrm{AC}2}$, the force at $f_{b}$, which is proportional to $\left(V_{\mathrm{AC}1}V_{\mathrm{AC}2}\right)$ as per Equation (12), can be used as the dominant excitation force. The ratio between the two amplitudes

$$\mu =\frac{V_{\mathrm{AC}2}}{V_{\mathrm{AC}1}}$$

is the signal modulation index.

The RLC circuit was tuned to set the electrical resonance frequency higher than that of the actuator $f_{e}>f_{m}$ using a small external inductor $L_{1}=60$ µH connected in series with actuator $I^{\prime }$. The resulting electrical resonance was measured as $f_{e}=6.27$ MHz using the technique described in Section 3.4.4. The carrier signal frequency was set to match the electrical resonance $f_{e}$, while the frequency of the baseband signal was swept up past the mechanical resonance $f_{m}$ in the range $f_{b}=\left[1.473-1.481\right]$ MHz over 100 ms.

**Figure 21:**
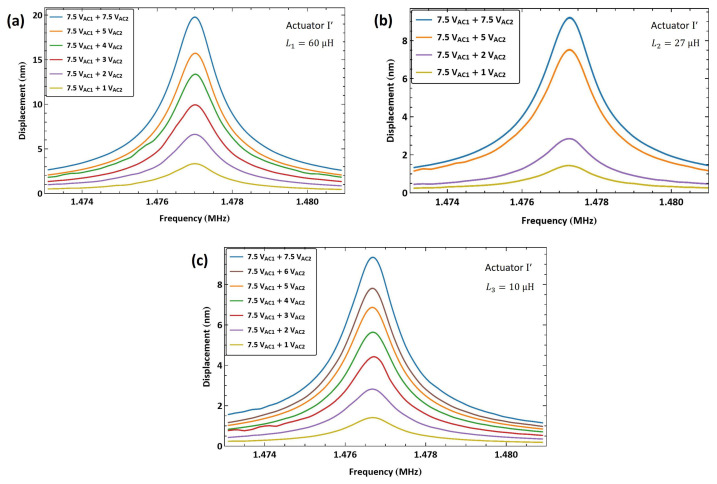
The frequency response of actuator $I^{\prime }$ tip displacement under excitation with an amplitude-modulated signal where the carrier frequency is set to (**a**) $f_{e}=6.27$ MHz, (**b**) $f_{e}=9.11$ MHz, and (**c**) $f_{e}=13.25$ MHz.

Figure 21a shows the frequency response of actuator $I^{\prime }$ tip displacement under six levels of the second voltage amplitude $V_{\mathrm{AC}2}$, while the amplitude of the first (amplifier) signal is held constant at $V_{\mathrm{AC}1}=7.5$ V. The maximum displacement realized was $w_{\mathrm{RMS}}=19.8$ nm observed at resonance $f_{m}=1.477$ MHz under a second voltage amplitude of $V_{\mathrm{AC}2}=7.5$ V (a modulation index of μ=1), which is 0.99% of the air gap.

To test the flexibility of this technique, we tuned the electrical resonance to a higher value by using a smaller external inductor $L_{2}=27$ µH. As a result, the electrical resonance increased to $f_{e}=9.11$ MHz. Figure 21b shows the frequency response of actuator $I^{\prime }$ under four levels of the second voltage amplitude $V_{\mathrm{AC}2}$ while the amplifier signal amplitude is held constant at $V_{\mathrm{AC}1}=7.5$ V. The maximum measured displacement was $w_{\mathrm{RMS}}=9.2$ nm for a voltage amplitude of $V_{\mathrm{AC}2}=7.5$ V, corresponding to 0.46% of the air gap.

A third inductor $L_{3}=10$ µH was also used with actuator $I^{\prime }$ to set the electrical resonance to $f_{e}=13.25$ MHz. Figure 21c shows the frequency response of actuator I$I^{\prime }$ under seven levels of the second signal voltage amplitude $V_{\mathrm{AC}2}$ while the amplifier signal amplitude is held constant at $V_{\mathrm{AC}1}=7.5$ V. The maximum measured displacement was $w_{\mathrm{RMS}}=9.3$ nm for a voltage amplitude of $V_{\mathrm{AC}2}=7.5$ V, corresponding to 0.47% of the air gap. The smaller displacements realized in the last two cases compared to the case of the first case, with the larger $L_{1}$ inductor, confirms our hypothesis above that the ESR of inductors at an µH level is negligible compared to the other circuit losses, which allows the larger inductance of $L_{1}$ to improve the overall circuit quality factor $Q_{e}$.

**Figure 22:**
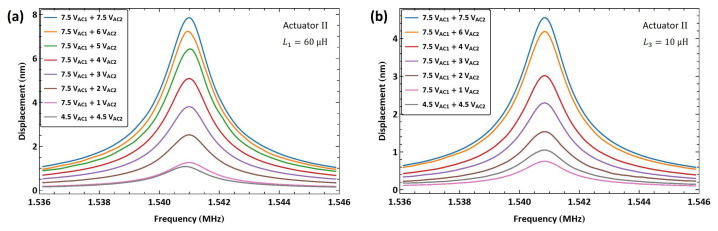
The frequency response of actuator II tip displacement under excitation with an amplitude-modulated signal where the carrier frequency is set to (**a**) $f_{e}=6.21$ MHz and (**b**) $f_{e}=13.1$ MHz.

We applied the same excitation technique to actuator II to verify the scheme repeatability. An external inductor $L_{1}=60$ µH was connected in series with the actuator, resulting in an electrical resonance at $f_{e}=6.21$ MHz. The difference between the electrical resonance in this case and that in actuator $I^{\prime }$ case is due to inter-inductor variability and variations in the circuit wiring. The baseband signal frequency was swept up in the range $f_{b}=\left[1.536-1.546\right]$ MHz. Figure 22a shows the frequency–response curves under eight levels of the second voltage amplitude $V_{\mathrm{AC}2}$. The maximum tip displacement achieved was $w_{\mathrm{RMS}}=7.9$ nm at resonance $f_{m}=1.5408$ MHz for a second voltage amplitude of $V_{\mathrm{AC}2}=7.5$ V, which is 0.395% of the air gap.

For comparison, the figure also incorporates a frequency–response curve with a lower amplifier voltage amplitude of $V_{\mathrm{AC}1}=4.5$ V and a second voltage amplitude of $V_{\mathrm{AC}2}=4.5$ V. It can be seen that the response in the latter case is comparable to that of the higher amplifier voltage under a much smaller second voltage of $V_{\mathrm{AC}2}=1$ V, thereby demonstrating the efficacy of the amplifier circuit.

The smaller $L_{3}=10$ µH inductor was connected in series with actuator II, resulting in an electrical resonance of $f_{e}=13.1$ MHz. Figure 22b shows the frequency–response curves of the tip displacement for seven levels of the second voltage amplitude $V_{\mathrm{AC}2}$. The largest measured RMS displacement was $w_{\mathrm{RMS}}=4.5$ nm at resonance for the second voltage amplitude of $V_{\mathrm{AC}2}=7.5$ V, corresponding to 0.23% of the air gap. The results also confirm that larger inductors $\left(L_{1}\right)$ result in higher amplification (larger $Q_{e}$) for µH-sized inductors.

Figure 22b also incorporates a frequency–response curve with a lower amplifier voltage amplitude of $V_{\mathrm{AC}1}=4.5$ V and a second voltage amplitude of $V_{\mathrm{AC}2}=4.5$ V. It can be seen that the response in this case falls between those for the smaller voltage amplitudes $V_{\mathrm{AC}2}=1$ V and 2 V and the higher amplifier voltage $V_{\mathrm{AC}2}=7.5$ V, further confirming the efficacy of the amplifier circuit.

#### 3.4.6. Comparison Among the Actuation Techniques

To evaluate the performance of the three actuation techniques and compare them, we define a magnification factor

(13) $$\mathrm{MF}=\frac{w_{\mathrm{RMS}}}{V_{\mathrm{RMS}}}\;\left(\mathrm{nm}/V\right)$$

that describes the efficiency of electromechanical coupling between the system input, namely the voltage signal applied to the MEMS circuit, and its output, the measured displacement w(t). To avoid the ambiguity posed by the presence of multiple local maxima in cases where either signal contains more than one frequency, those signals are quantified in terms of their RMS. The magnification factor serves as a figure of merit, allowing us to quantify the relative merits of various actuation schemes in various applications.

**Figure 23:**
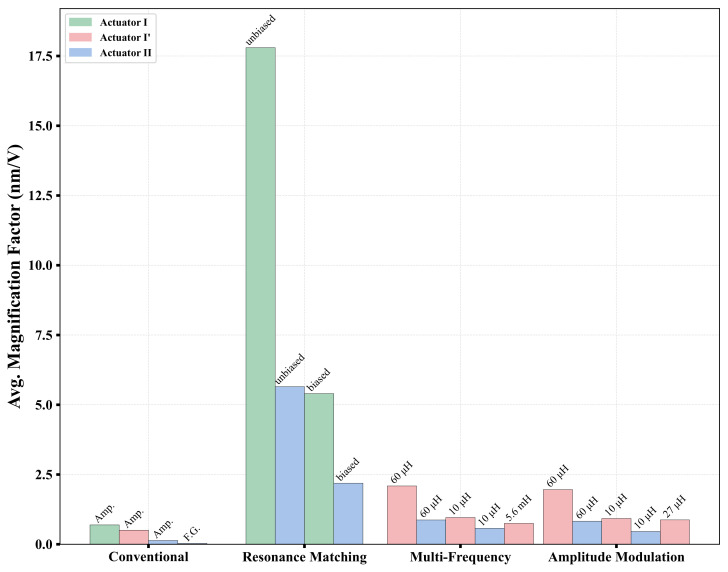
Comparison of the average magnification factors for different actuation techniques.

The magnification factors realized for actuators I, $I^{\prime }$, and II are listed in Table S1, Table S2, and Table S3, respectively, in the Supplementary Materials. Comparison across the various actuation schemes, as shown in Figure 23, shows that unbiased resonance matching is most effective in achieving the highest magnification factors, ${\mathrm{MF}}_{\mathrm{Avg}}=17.8$ nm/V and ${\mathrm{MF}}_{\mathrm{Avg}}=5.7$ nm/V compared to magnification factors of ${\mathrm{MF}}_{\mathrm{Avg}}=5.4$ nm/V and ${\mathrm{MF}}_{\mathrm{Avg}}=2.2$ nm/V for the biased cases in actuators I and II, respectively. This is expected since unbiased resonance matching amplifies input voltage by $Q_{e}^{2}$ compared an amplification by $Q_{e}$ only in the biased case.

The performance of multi-frequency and amplitude modulated actuations is inferior to biased resonance matching, but they are similar to each other with a slight advantage to the former. The advantage of biased resonance matching is due to its actuation voltage benefiting from amplification via the $Q_{e}$ of the RLC circuit and the bias voltage $V_{\mathrm{DC}}$, while the latter two realize their amplification only through the $Q_{e}$ of the RLC circuit. In both multi-frequency and amplitude modulated actuations, lower electrical resonance frequencies $f_{e}$ (larger inductors) were found to realize better magnification factors. Actuator $I^{\prime }$ achieved ${\mathrm{MF}}_{\mathrm{Avg}}=2.089$ nm/V at lower frequency, $f_{e}=6.27$ MHz, compared to ${\mathrm{MF}}_{\mathrm{Avg}}=0.960$ nm/V at higher frequency, $f_{e}=13.25$ MHz. Similarly, actuator II exhibited an improvement from ${\mathrm{MF}}_{\mathrm{Avg}}=0.575$ nm/V at higher frequency ,$f_{e}=13.1$ MHz, to ${\mathrm{MF}}_{\mathrm{Avg}}=0.871$ nm/V at lower frequency, $f_{e}=6.21$ MHz. For amplitude modulation, a similar trend was observed, with actuator $I^{\prime }$ achieving ${\mathrm{MF}}_{\mathrm{Avg}}=1.956$ nm/V at lower $f_{e}$ versus ${\mathrm{MF}}_{\mathrm{Avg}}=0.925$ nm/V at a higher $f_{e}$. Actuator II followed the same pattern, with ${\mathrm{MF}}_{\mathrm{Avg}}$ improving from 0.460 nm/V at higher $f_{e}$ to 0.826 nm/V at lower $f_{e}$.

Finally, we observe that the magnification factor of actuator I under voltage amplifier actuation was ${\mathrm{MF}}_{\mathrm{Avg}}=0.694$ nm/V compared to ${\mathrm{MF}}_{\mathrm{Avg}}=0.136$ nm/V for actuator II under the same actuation scheme. The discrepancy in the magnification factor between the two actuators is probably due to the higher stiffness of actuator II.

## 4. Conclusions

The relative merits of three resonant drive methods have been investigated and compared with a view to reducing the high voltage requirements of electrostatic MEMS actuators. As a ground truth measure, the three methods were compared to conventional actuation using a function generator and voltage amplifier. Furthermore, a coupled electromechanical model was introduced to interpret the response of the actuator in conjunction with the resonant drive circuit.

We found that resonant circuits are more effective at amplifying the MEMS response than voltage amplifiers in the high frequency range (>1 MHz). At lower frequencies, voltage amplifiers are more flexible and stable, whereas resonant circuits suffered larger losses due to the associated parasitic capacitance. Moreover, we found that multi-frequency and amplitude modulation techniques are more flexible and easier to adjust compared to resonance matching. However, resonance matching is more effective for unbiased (AC) excitation as it results in higher voltage amplification (proportional to $Q_{e}^{2}$) than the other two techniques or resonance matching with a biased signal, where voltage amplification was only proportional to $Q_{e}$. We also conclude that using biased signals with any of the resonant drive techniques is ineffective for the purposes of amplification. While it can be used to tune the MEMS nonlinearity, it should be noted that it may also result in dielectric charging.

**Figure 24:**
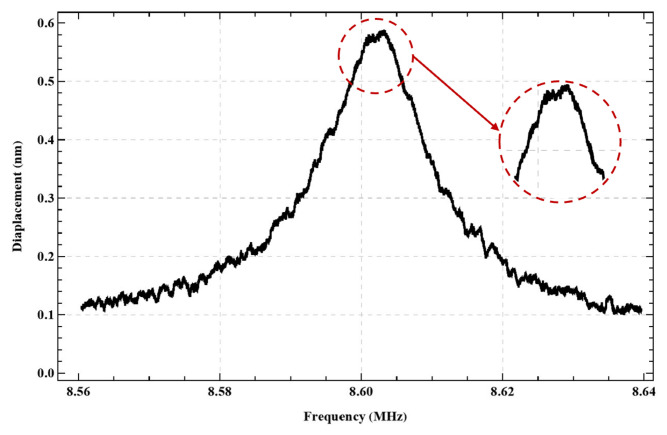
Frequency response of the second out-of-plane bending mode of actuator $I^{\prime }$.

The inefficiency of resonant circuits at low frequencies meant that actuation using the additive version of the multi-frequency excitation technique $\left(f_{1}+f_{2}\right)$ proved ineffective in the case of the actuator under study. However, this version of the multi-frequency excitation technique should be practical for higher-frequency actuators. For example, we employed it to locate the second out-of-plane bending mode of actuator $I^{\prime }$ shown in Figure 24. We found indications of an interaction between the second in-plane and out-of-plane bending modes under the additive version of multi-frequency excitation with an external inductor L=130 µH, where the first signal frequency was set to $f_{1}=f_{e}=4.2$ MHz and the second signal frequency was swept in the range $f_{2}=4.36-4.44$ MHz. We observed two peaks in the frequency response of actuator $I^{\prime }$ within this frequency range, suggesting the presence of two distinct vibration modes.

Nevertheless, multi-frequency excitation presents additional challenges compared to the other two techniques, particularly in signal generation and system implementation. Unlike amplitude modulation, where the carrier frequency $f_{e}$ provides a fixed reference for the baseband signal $f_{b}$, multi-frequency signal generation requires each frequency component to be independently generated and precisely tuned.

Although both amplitude-modulated and multi-frequency signals have multiple frequency components, their implementation differs significantly. The former is often simpler, as it can be achieved using a single DDS with internal programmable amplitude control (e.g., AD9854). In contrast, generating a stable multi-frequency signal typically requires either two separate DDS chips or a multi-channel DDS (e.g., AD9959), along with a summation circuit to combine the signals, increasing the system complexity.

While we assumed in our comparison that the amplification of the RLC circuit is constant and equal to the electrical quality factor $Q_{e}$ across the entire frequency range of interest, this assumption holds only when the mechanical quality factor is much larger than the electrical quality factor $\left(Q_{m}>>Q_{e}\right)$ [25]. This was true in the actuators used in this study but is not in general true. Where the two quality factors are of the same order, they would continue to be decoupled under multi-frequency and amplitude modulation techniques, but the amplification factor will vary over the relevant frequency range (half-power bandwidth) of the MEMS under the resonance matching technique.

Other limitations of resonant circuits include the presence of parasitic capacitance and inductance, which can significantly mistune the circuit and require online tuning. Parasitic capacitance can also introduce signal leakage and crosstalk and degrade the electrical quality factor. Additionally, as frequency increases, the signal may approach the system’s cutoff frequency, beyond which the signal will attenuate.

Furthermore, the skin effect becomes more pronounced at high frequencies, causing AC currents to concentrate near the conductor’s surface. This increases resistance, resulting in additional losses that degrade the performance [39]. Similarly, on-chip inductors typically exhibit lower quality factors (Q<10) compared to off-chip inductors.

## Figures and Tables

**Table 1:** Actuator dimensions.

Parameter	µm
beam length (*l*)	86
beam width (*b*)	10
beam thickness (*h*)	10
capacitive gap (*d*)	2
Metal pad thickness ($h_{p}$)	1

## Data Availability

The raw data supporting the conclusions of this article will be made available by the authors on request.

## Supplementary Materials

The following supporting information can be downloaded at https://www.mdpi.com/article/10.3390/s25061719/s1.

## References

[1] Kaajakari V. (2009). Practical MEMS.

[2] Younis M.I. (2011). MEMS Linear and Nonlinear Statics and Dynamics.

[3] Shama Y.S., Rahmanian S., Mouharrar H., Abdelrahman R., Elhady A., Abdel-Rahman E.M. (2024). Unraveling the Nature of Sensing in Electrostatic MEMS Gas Sensors. Microsyst. Nanoeng..

[4] Pengwang E., Rabenorosoa K., Rakotondrabe M., Andreff N. (2016). Scanning Micromirror Platform Based on MEMS Technology for Medical Application. Micromachines.

[5] Huang X.M.H., Manolidis M., Jun S.C., Hone J. (2005). Nanomechanical Hydrogen Sensing. Appl. Phys. Lett..

[6] Li E., Jian J., Yang F., Ma Z., Hao Y., Chang H. (2024). Characterization of Sensitivity of Time Domain MEMS Accelerometer. Micromachines.

[7] Chen L.-T., Lee C.-Y., Cheng W.-H. (2008). MEMS-Based Humidity Sensor with Integrated Temperature Compensation Mechanism. Sens. Actuators A Phys..

[8] Asri M.I.A., Hasan M.N., Fuaad M.R.A., Yunos Y.M., Ali M.S.M. (2021). MEMS Gas Sensors: A Review. IEEE Sens. J..

[9] Mi J., Wang Q., Han X. (2024). Low-Cost MEMS Gyroscope Performance Improvement under Unknown Disturbances through Deep Learning-Based Array. Sens. Actuators A Phys..

[10] Sattler R., Plötz F., Fattinger G., Wachutka G. (2002). Modeling of an electrostatic torsional actuator: Demonstrated with an RF MEMS switch. Sens. Actuators A Phys..

[11] Hossain M.I., Zahid M.S., Chowdhury M.A., Hossain M.M.M., Hossain N. (2023). MEMS-Based Energy Harvesting Devices for Low-Power Applications—A Review. Results Eng..

[12] Chuang W.-C., Lee H.-L., Chang P.-Z., Hu Y.-C. (2010). Review on the Modeling of Electrostatic MEMS. Sensors.

[13] Zhang W.-M., Yan H., Peng Z.-K., Meng G. (2014). Electrostatic Pull-In Instability in MEMS/NEMS: A Review. Sens. Actuators A Phys..

[14] Algamili A.S., Khir M.H.M., Dennis J.Q., Ahmed A.Y., Alabsi S.S., Ba Hashwan S.S., Junaid M.M. (2021). A Review of Actuation and Sensing Mechanisms in MEMS-Based Sensor Devices. Nanoscale Res. Lett..

[15] Jaber N., Ramini A., Carreno A.A.A., Younis M.I. (2016). Higher order modes excitation of electrostatically actuated clamped–clamped microbeam experimental and analytical investigation. J. Micromech. Microeng..

[16] Nayfeh A.H., Younis M.I. (2005). Dynamics of MEMS resonators under superharmonic and subharmonic excitations. J. Micromech. Microeng..

[17] Shama Y.S., Abdelrahman R., Arabi M., Saritas R., Rahmanian S., Gulsaran A., Kocer S., Elhady A., Mouharrar H., Abdel- Rahman E.M., Lacarbonara W. (2024). A Comparative Study of Two Types of Bifurcation-Based MEMS Sensors. Advances in Nonlinear Dynamics, Volume III, Proceedings of the ICNDA 2023, NODYCON Conference.

[18] Park S., Bai Y., Yeow J.T.W. Design and Analysis of Resonant Drive Circuit for Electrostatic Actuators. Proceedings of the 2010 International Symposium on Optomechatronic Technologies.

[19] Park S., Abdel-Rahman E. Low Voltage Electrostatic Actuation and Displacement Measurement Through Resonant Drive Circuit. Proceedings of the ASME 2012 International Design Engineering Technical Conferences & Computers and Information in Engineering Conference.

[20] Park S., Pallapa M., Yeow J.T.W., Abdel-Rahman E. Low Voltage Electrostatic Actuation and Angular Displacement Measurement of Micromirror Coupled with Resonant Drive Circuit. Proceedings of the IECON 2012—38th Annual Conference on IEEE Industrial Electronics Society.

[21] Park S., Khater M., Abdel-Rahman E. Low Voltage Electrostatic Actuation for MEMS Actuator Using Frequency Modulation. Proceedings of the ASME 2013 International Design Engineering Technical Conferences and Computers and Information in Engineering Conference.

[22] Chung S.-R., Abdel-Rahman E.M., Yeow J. A MEMS Analog Demodulator. Proceedings of the IECON 2012—38th Annual Conference on IEEE Industrial Electronics Society.

[23] Chung S.R., Park S., Abdel-Rahman E.M., Yeow J.T., Khater M. (2013). Architecture for MEMS-based analogue demodulation. J. Micromech. Microeng..

[24] Cady W.G. (1922). The Piezo-Electric Resonator. Proc. Inst. Radio Eng..

[25] Truitt P.A., Hertzberg J.B., Huang C.C., Ekinci K.L., Schwab K.C. (2007). Efficient and Sensitive Capacitive Readout of Nanomechanical Resonator Arrays. Nano Lett..

[26] Alsaleem F.M., Hasan M.H. A Novel Low Voltage Electrostatic MEMS Resonator Sensor Based on Double Resonance Dynamic Amplification. Proceedings of the ASME 2017 Dynamic Systems and Control Conference.

[27] Hasan M.H., Alsaleem F.M., Jaber N., Hafiz M.A.A., Younis M.I. (2018). Simultaneous Electrical and Mechanical Resonance Drive for Large Signal Amplification of Micro Resonators. Aip Adv..

[28] Jaber N., Hafiz M.A.A., Kazmi S.N.R., Younis M.I. (2019). Efficient Excitation of Micro/Nano Resonators and Their Higher Order Modes. Sci. Rep..

[29] Ouakad H.M., Hasan M.H., Jaber N.R., Hafiz M.A.A., Alsaleem F., Younis M. (2020). On the Double Resonance Activation of Electrostatically Actuated Microbeam-Based Resonators. Int. J. Non-Linear Mech..

[30] Cowen A., Hames G., Glukh K., Hardy B. (2014). PiezoMUMPs™ Design Handbook.

[31] Kambali P.N., Pandey A.K. (2016). Capacitance and Force Computation Due to Direct and Fringing Effects in MEMS/NEMS Arrays. IEEE Sensors J..

[32] Polytec Inc UHF-120 Ultra High Frequency Vibrometer Datasheet. www.polytec.com.

[33] Liquid Instruments Moku:Pro Datasheet. www.liquidinstruments.com.

[34] Tabor Electronics Ltd 400Vp-p Four Channel Signal Amplifier Model 9400 Datasheet. www.taborelec.com/9400.

[35] Polytec Inc Polytec Scanning Vibrometer Software Manual. Windows..

[36] Elhady A., Alghamdi M.S., Abdel-Rahman E. (2023). Experimental construction of force- and frequency-response curves of nonlinear resonators. Chaos.

[37] Ertasgin G., Whaley D.M. (2024). Analysis and Optimization of Output Low-Pass Filter for Current-Source Single-Phase Grid-Connected PV Inverters. Appl. Sci..

[38] Polytec Inc OFV-5000 Vibrometer Controller User Manual. www.polytec.com.

[39] Giacoletto L.J. (1996). Frequency-and Time-Domain Analysis of Skin Effects. IEEE Trans. Magn..

