# Supplementary material for: Resonant Drive Techniques for Electrostatic Microelectromechanical Systems (MEMS): A Comparative Study
Source: Sensors (Basel). 2025 Mar 10;25(6):1719. doi: 10.3390/s25061719 (PMC11944924; doi:10.3390/s25061719)
Supplement: Supplementary file 1 [file sensors-25-01719-s001.zip › sensors-3460470-supplementary.pdf]

Supplementary Material for

Resonant Drive Techniques for MEMS: A Comparative Study

Rana Abdelrahman[1, 3] and Alaaeldin Elhady[1] Yasser S. Shama[1, 3, 4] Mohamed Abdelrahman[1, 3] Alexis Jollivet[5] Dogu Ozyigit[2, 3] Mustafa Yavuz[2, 3] Eihab M. Abdel-Rahman[1, 3]

[1]Systems Design Engineering, University of Waterloo, 200 University Ave W, Waterloo.

[2]Mechanical and Mechatronics Engineering Department, University of Waterloo, Waterloo, ON N2L 3G1.

[3]Waterloo Institute for Nanotechnology (WIN), University of Waterloo, Waterloo, ON N2L 3G1, Canada.

[4]Mechanical Engineering, Benha Faculty of Engineering, Benha University

[5]Electronics and Digital Technology, Polytech Nantes, University of Nantes, France

1 Realized Magnification Factors

The magnification factors for actuators I, I′, and II were determined based on the measured RMS displacement response relative to the RMS value of the applied excitation MF = $\frac{w_{\mathrm{RMS}}}{V_{\mathrm{RMS}}}$ (nm/V).

Table S1: Realized magnification factors for actuator I.

V_{in} (V)	V_{RMS} (V)	w_{RMS} (nm)	MF (nm/V)	$\mathrm{MF}_{\mathrm{Avg}} \pm \sigma$ (nm/V)
Voltage Amplifier				
114 V_{AC}	80.61	70.3	0.868	0.694 ± 0.201
105 V_{AC}	74.25	61.0	0.822	
90 V_{AC}	63.64	42.5	0.660	
60 V_{AC}	42.43	17.9	0.424	
Resonance Matching (unbiased)				
10 V_{AC}	7.07	189.4	26.780	17.839 ± 8.971
8 V_{AC}	5.66	126.6	22.388	
6 V_{AC}	4.24	68.2	16.083	
4 V_{AC}	2.83	17.3	6.104	
Resonance Matching (biased)				
31.5 V_{DC} + 10 V_{AC}	32.28	171.6	5.316	5.415 ± 0.277
25 V_{DC} + 10 V_{AC}	25.98	135.1	5.201	
20 V_{DC} + 10 V_{AC}	21.21	121.5	5.727	

Table S2: Realized magnification factors actuator I′.

V_{in} (V)	V_{RMS} (V)	w_{RMS} (nm)	MF (nm/V)	$\mathrm{MF}_{\mathrm{Avg}} \pm \sigma$ (nm/V)
Voltage Amplifier				
108 V_{AC}	76.37	72.9	0.954	0.505 ± 0.335
105 V_{AC}	74.25	69.4	0.935	
102 V_{AC}	72.13	63.9	0.886	
99 V_{AC}	70.00	60.2	0.860	

V_{in} (V)	V_{RMS} (V)	w_{RMS} (nm)	MF (nm/V)	$MF_{Avg} \pm \sigma$ (nm/V)
51 V_{AC}	36.06	15.9	0.442	
45 V_{AC}	31.82	12.5	0.394	
39 V_{AC}	27.58	10.1	0.366	
24 V_{AC}	16.97	3.8	0.224	
21 V_{AC}	14.85	2.9	0.192	
18 V_{AC}	12.73	2.1	0.165	
15 V_{AC}	10.61	1.5	0.140	
Multi-Frequency Excitation (60 µH)				
5 V_{AC1} + 5 V_{AC2}	5.00	15.1	3.016	2.089 ± 0.883
5 V_{AC1} + 4 V_{AC2}	4.53	12.5	2.770	
5 V_{AC1} + 3 V_{AC2}	4.12	9.2	2.223	
5 V_{AC1} + 2 V_{AC2}	3.81	6.0	1.579	
5 V_{AC1} + 1 V_{AC2}	3.61	3.1	0.857	
Multi-Frequency Excitation (10 µH)				
7.5 V_{AC1} + 7.5 V_{AC2}	7.50	9.1	1.213	0.960 ± 0.317
7.5 V_{AC1} + 6 V_{AC2}	6.79	8.3	1.218	
7.5 V_{AC1} + 5 V_{AC2}	6.37	7.5	1.183	
7.5 V_{AC1} + 4 V_{AC2}	6.01	6.5	1.087	
7.5 V_{AC1} + 3 V_{AC2}	5.71	5.4	0.945	
7.5 V_{AC1} + 2 V_{AC2}	5.49	3.8	0.695	
7.5 V_{AC1} + 1 V_{AC2}	5.35	2.0	0.380	
Multi-Frequency Excitation (5.6 mH)				
4.5 V_{AC1} + 4.5 V_{AC2}	4.50	4.7	1.050	0.749 ± 0.260
4.5 V_{AC1} + 3.5 V_{AC2}	4.03	3.4	0.846	
4.5 V_{AC1} + 2.5 V_{AC2}	3.64	2.4	0.660	
4.5 V_{AC1} + 1.5 V_{AC2}	3.35	1.5	0.440	
Amplitude Modulation (60 µH)				
7.5 V_{AC1} + 7.5 V_{AC2}	6.50	19.8	3.044	1.956 ± 0.916
7.5 V_{AC1} + 5 V_{AC2}	5.86	15.7	2.681	
7.5 V_{AC1} + 4 V_{AC2}	5.67	13.4	2.358	

V_{in} (V)	V_{RMS} (V)	w_{RMS} (nm)	MF (nm/V)	$MF_{Avg} \pm \sigma$ (nm/V)
7.5 V_{AC1} + 3 V_{AC2}	5.51	9.9	1.803	
7.5 V_{AC1} + 2 V_{AC2}	5.40	6.6	1.227	
7.5 V_{AC1} + 1 V_{AC2}	5.33	3.3	0.625	
Amplitude Modulation (10 µH)				
7.5 V_{AC1} + 7.5 V_{AC2}	6.50	9.3	1.439	0.925 ± 0.422
7.5 V_{AC1} + 6 V_{AC2}	6.09	7.8	1.282	
7.5 V_{AC1} + 5 V_{AC2}	5.86	6.9	1.171	
7.5 V_{AC1} + 4 V_{AC2}	5.67	5.6	0.995	
7.5 V_{AC1} + 3 V_{AC2}	5.51	4.4	0.803	
7.5 V_{AC1} + 2 V_{AC2}	5.40	2.8	0.523	
7.5 V_{AC1} + 1 V_{AC2}	5.33	1.4	0.265	
Amplitude Modulation (27 µH)				
7.5 V_{AC1} + 7.5 V_{AC2}	6.50	9.2	1.416	0.874 ± 0.562
7.5 V_{AC1} + 5 V_{AC2}	5.86	7.5	1.282	
7.5 V_{AC1} + 2 V_{AC2}	5.40	2.8	0.527	
7.5 V_{AC1} + 1 V_{AC2}	5.33	1.4	0.270	

Table S3: Realized magnification factors actuator II.

V_{in} (V)	V_{RMS} (V)	w_{RMS} (nm)	MF (nm/V)	$MF_{Avg} \pm \sigma$ (nm/V)
Function Generator				
10 V_{AC}	7.07	0.257	0.0364	0.0332 ± 0.0045
8 V_{AC}	5.66	0.170	0.0301	
Voltage Amplifier				
57 V_{AC}	40.31	8.4	0.208	0.136 ± 0.044
48 V_{AC}	33.94	5.8	0.162	
45 V_{AC}	31.82	5.2	0.162	
39 V_{AC}	27.57	3.8	0.138	
33 V_{AC}	23.33	2.9	0.122	
30 V_{AC}	21.21	2.5	0.118	

V_{in} (V)	V_{RMS} (V)	w_{RMS} (nm)	MF (nm/V)	$MF_{Avg} \pm \sigma$ (nm/V)
21 V_{AC}	14.85	1.1	0.0763	
15 V_{AC}	10.61	0.590	0.0896	
Multi-Frequency Excitation (60 μH)				
7.5 V_{AC1} + 7.5 V_{AC2}	7.50	8.7	1.16	
7.5 V_{AC1} + 6 V_{AC2}	6.792	8.0	1.18	
7.5 V_{AC1} + 3 V_{AC2}	5.712	5.7	0.998	0.871 ± 0.354
7.5 V_{AC1} + 2 V_{AC2}	5.489	3.7	0.668	
7.5 V_{AC1} + 1 V_{AC2}	5.35	1.9	0.352	
Multi-Frequency Excitation (10 μH)				
7.5 V_{AC1} + 6 V_{AC2}	6.79	5.1	0.747	
7.5 V_{AC1} + 5 V_{AC2}	6.37	4.6	0.728	
7.5 V_{AC1} + 4 V_{AC2}	6.01	4.1	0.685	0.575 ± 0.195
7.5 V_{AC1} + 3 V_{AC2}	5.71	3.4	0.589	
7.5 V_{AC1} + 2 V_{AC2}	5.49	2.5	0.464	
7.5 V_{AC1} + 1 V_{AC2}	5.35	1.3	0.238	
Amplitude Modulation (60 μH)				
7.5 V_{AC1} + 7.5 V_{AC2}	6.50	7.9	1.21	
7.5 V_{AC1} + 6 V_{AC2}	6.09	7.2	1.19	
7.5 V_{AC1} + 5 V_{AC2}	5.86	6.4	1.1	
7.5 V_{AC1} + 4 V_{AC2}	5.67	5.1	0.897	0.826 ± 0.376
7.5 V_{AC1} + 3 V_{AC2}	5.51	3.8	0.69	
7.5 V_{AC1} + 2 V_{AC2}	5.40	2.5	0.467	
7.5 V_{AC1} + 1 V_{AC2}	5.33	1.3	0.237	
Amplitude Modulation (10 μH)				
7.5 V_{AC1} + 7.5 V_{AC2}	6.50	4.6	0.701	
7.5 V_{AC1} + 6 V_{AC2}	6.09	4.2	0.687	
7.5 V_{AC1} + 4 V_{AC2}	5.67	3.0	0.532	0.460 ± 0.223
7.5 V_{AC1} + 3 V_{AC2}	5.51	2.3	0.417	
7.5 V_{AC1} + 2 V_{AC2}	5.40	1.5	0.285	
7.5 V_{AC1} + 1 V_{AC2}	5.33	0.753	0.141	
